# Analysis of Microarray-Identified Genes and MicroRNAs Associated with Idiopathic Pulmonary Fibrosis

**DOI:** 10.1155/2017/1804240

**Published:** 2017-05-14

**Authors:** Lichao Fan, Xiaoting Yu, Ziling Huang, Shaoqiang Zheng, Yongxin Zhou, Hanjing Lv, Yu Zeng, Jin-Fu Xu, Xuyou Zhu, Xianghua Yi

**Affiliations:** ^1^Department of Pathology, Tongji Hospital, Tongji University School of Medicine, Shanghai 200065, China; ^2^Department of Respiratory and Critical Care Medicine, Shanghai Pulmonary Hospital, Tongji University School of Medicine, Shanghai 200443, China; ^3^Department of Radiology, Tongji Hospital, Tongji University School of Medicine, Shanghai 200065, China; ^4^Department of Thoracic-Cardiovascular Surgery, Tongji Hospital, Tongji University School of Medicine, Shanghai 200065, China; ^5^Department of Respiratory Medicine, Shanghai Tongji Hospital, Tongji University School of Medicine, Shanghai 200065, China

## Abstract

The aim of this study was to identify potential microRNAs and genes associated with idiopathic pulmonary fibrosis (IPF) through web-available microarrays. The microRNA microarray dataset GSE32538 and the mRNA datasets GSE32537, GSE53845, and GSE10667 were downloaded from the Gene Expression Omnibus (GEO) database. Differentially expressed miRNAs (DE-miRNAs)/genes (DEGs) were screened with GEO2R, and their associations with IPF were analyzed by comprehensive bioinformatic analyses. A total of 45 DE-microRNAs were identified between IPF and control tissues, whereas 67 common DEGs were determined to exhibit the same expression trends in all three microarrays. Furthermore, functional analysis indicated that microRNAs in cancer and ECM-receptor interaction were the most significant pathways and were enriched by the 45 DE-miRNAs and 67 common DEGs. Finally, we predicted potential microRNA-target interactions between 17 DE-miRNAs and 17 DEGs by using at least three online programs. A microRNA-mediated regulatory network among the DE-miRNAs and DEGs was constructed that might shed new light on potential biomarkers for the prediction of IPF progression.

## 1. Introduction

Idiopathic pulmonary fibrosis (IPF), which is the most common form of the idiopathic interstitial pneumonias (IIPs), is characterized by clinical symptoms of cough and dyspnea, restrictive pulmonary function with impaired gas exchange, and progressive lung scarring [1]. Recently, two modestly effective drugs for treating IPF have been identified [2, 3]. However, the prognosis of IPF remains grave, thus emphasizing a need for a more complete understanding of its mechanisms of disease pathogenesis.

In the past decades, a number of studies have revealed that microarrays can be used to identify potential biomarkers in numerous diseases at molecular level with more effective and detailed insights [4, 5]. MicroRNAs (miRNA) are a class of noncoding RNAs that have drawn considerable attention for their critical effects in cellular processes such as apoptosis, proliferation, and differentiation. Over the last decade, more and more studies have been performed to find potential biomarkers for the prediction of IPF. Through microarray profiles, IPF has been reported to be interrelated with multiple putative miRNAs, including miR-92a [6], miR-210 [7], miR-29 [8], miR-326 [9], miR-98 [10], and miR-let-7d [11]. However, only a very small number of differentially expressed genes were found and they were not consistent across all these studies. Therefore, further development into clinically useful biomarkers and therapeutic targets were limited by these incongruous results. It has been well recognized that small sample sizes, different microarray platforms, and different statistical methods are among the limiting factors contributed to the discordant results. To resolve this limitation, meta-analysis represents a powerful approach to combine different datasets from different studies to improve the reliability and generalizability of the findings by increasing its statistical power analysis. Meta-analysis on gene expression data has yielded new biological insights, as well as identification of more robust and reliable candidate biomarkers and therapeutic targets [12, 13].

The present study aimed to identify differentially expressed miRNAs (DE-miRNAs) in microRNA expression profiles and differentially expressed genes (DEGs) in three messenger RNA (mRNA) expression profiles through the Gene Expression Omnibus (GEO) database to explore the biological processes in IPF. Correlations between the DEGs and DE-miRNAs were examined using comprehensive bioinformatics analysis. We combined the information retrieved from the DE-miRNA and DEG data, PPI interaction network construction, and pathway enrichment analysis and then screened out potential biomarkers for IPF. In addition, text mining was conducted to obtain ideas and clues for further experimental research, assisting in indicating more biomarkers of IPF.

## 2. Materials and Methods

### 2.1. Acquisition and Analysis of Datasets

Microarray data from IPF-related microRNA and mRNA expression profiles were retrieved and downloaded from the National Center for Biotechnology Information (NCBI) GEO database (http://www.ncbi.nlmNih.gov/geo). Queries were performed using “IPF” as a keyword. The search was restricted to the following specific fields: study type, expression profiling by array, and species—*Homo sapiens*. We downloaded the microRNA expression microarray dataset GSE32538 [14] and the mRNA expression microarray datasets GSE32537 [14], GSE53845 [15], and GSE10667 [16–18].

### 2.2. Inclusion Criteria for Differentially Expressed MicroRNAs and Genes

GEO2R (http://www.ncbi.nlm.nih.gov/geo/geo2r/), a web tool, can perform sophisticated R-based analyses of GEO data and presents the results as a table of differential gene expression that can be visualized using GEO Profile graphics [19]. This tool is based on a *t*-test (ANOVA) or analysis of variance, and it is useful for comparing two or more groups of samples across the same experimental conditions to characterize differentially expressed microRNAs or genes. In the present study, microRNAs and genes that were differentially expressed between IPF and controls were screened using an adjusted *p* value (adj. *p*) of less than 0.05 and a |log fold change| of >1.0 as thresholds. DE-miRNAs or DEGs that were common to at least two expression profile datasets were selected using the Bioinformatics & Research Computing website (http://jura.wi.mit.edu/bioc/tools/compare.php). Bioinformatic analyses of the DE-miRNAs and DEGs were conducted. Pathway enrichment analysis of differentially expressed microRNAs was performed using DIANA miRPath (http://diana.imis.athenainnovation.gr/DianaTools/index.php?r=mirpath/index) [20]. DAVID (http://david.abcc.ncifcrf.gov/) was used to analyze the pathway enrichment of the differentially expressed genes [21]. Protein/gene interactions were analyzed using STRING (http://www.string.embl.de/) [22], and mRNA-microRNA interactions were analyzed using the miRanda (http://www.microrna.org/microrna/home.do) [23], picTar (http://pictar.mdc-berlin.de/) [24], TargetScan (http://www.targetscan.org/) [25], PITA (http://genie.weizmann.ac.il/pubs/mir07/mir07_data.html/) [26], and RNA22 (http://cbcsrv.watson.ibm.com/ma 22 .html) [27] tools.

## 3. Results

### 3.1. Microarray Datasets That Met the Inclusion Criteria

In the present study, the microRNA expression profiling dataset was GSE32538, which was generated by the University of Colorado, Anschutz Medical Campus using the GPL8786 Affymetrix miRNA Array platform. The samples used to generate the GSE32538 dataset were lung tissues from 167 subjects with IIP and 50 nondiseased controls. The mRNA expression profiling datasets studied were GSE32537, GSE53845, and GSE10667. GSE32537 was generated by the University of Colorado, Anschutz Medical Campus using the GPL6244 Affymetrix Human Gene 1.0 ST Array platform. The data were derived from 167 subjects with IIP and 50 nondiseased controls. GSE53845 originated from Genentech, Inc., using the GPL6480 Agilent-014850 Whole Human Genome Microarray platform. The data were derived from lung tissue samples from 40 IPF patients and 8 healthy controls. The GSE10667 dataset was generated by the University of Pittsburgh using the GPL4133 Agilent-014850 Whole Human Genome Microarray platform. The data were derived from 23 IPF and 15 control lung tissue samples (Table 1).

### 3.2. Differentially Expressed MicroRNAs and Pathway Enrichment

**Figure 1:**
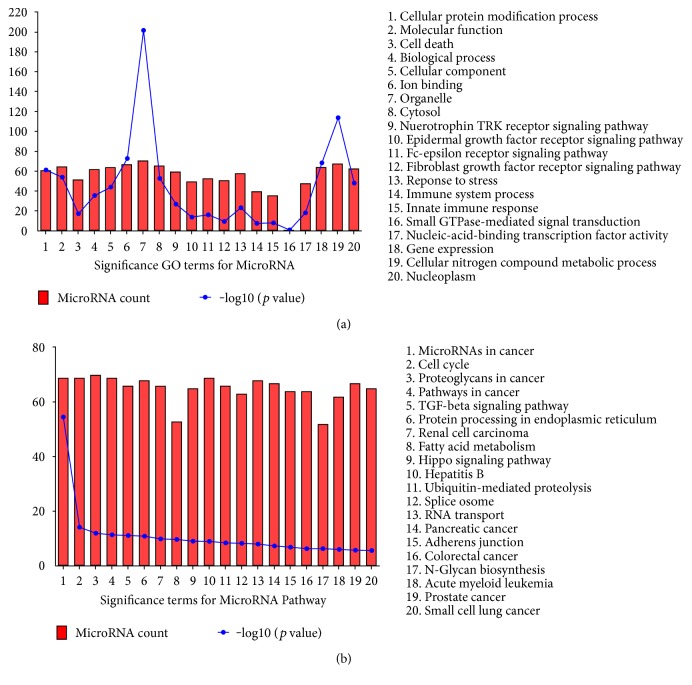
Significant GO terms and pathway analysis obtained from the miRNA expression datasets. (a) Significant GO terms for DE-miRNAs. (b) Significant terms for De-miRNA pathways.

Differentially expressed microRNAs of the IPF and control groups in the GES32538 expression profiling datasets were analyzed using the GEO2R tool. After rigorous screening using adj. *p* < 0.05 and |logFC| > 1, a total of 45 differentially expressed miRNAs were identified in the IPF group compared with those in the control group. Three microRNAs (has-miR-205, has-miR-34c, has-miR-31) were significantly upregulated in IPF, whereas the expression levels of the remaining 42 microRNAs were decreased. The top 10 dysregulated miRNAs were hsa-miR-205, has-miR-34c-3p, hsa-miR-34c-5p, hsa-miR-31, hsa-miR-532-5p, hsa-miR-652, hsa-miR-130a, hsa-miR-210, hsa-miR-500, and hsa-miR-193a-5p (Table 2). Pathway enrichment analysis was performed on these 45 differentially expressed microRNAs using DIANA miRPath [20]. A total of 82 signaling pathways were identified (*p* < 0.05), and the 20 most significantly enriched pathways were selected according to their *p* values (Figure 1(a)). Functional analysis demonstrated that the dysregulated miRNAs could be enriched into 263 functional GO terms, and the top 20 are shown in Figure 1(b). Heatmap select pathway intersections (with 2 or more miRNAs) are shown in Supplementary Figure 1 available online at https://doi.org/10.1155/2017/1804240.

### 3.3. Screening for Differentially Expressed Genes in Three Sets of mRNA Microarrays and Analysis of the Correlation between Differentially Expressed Genes in IPF

**Figure 2:**
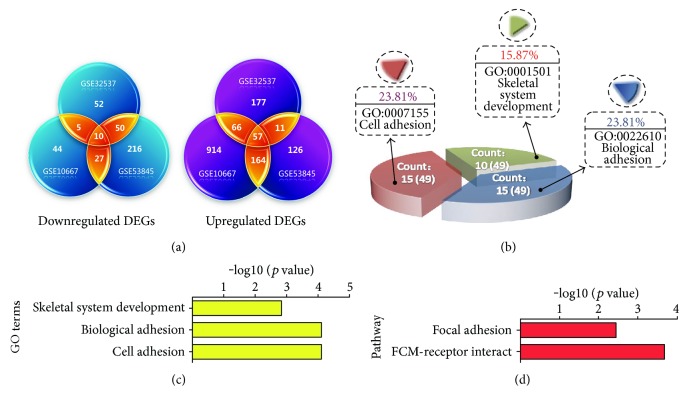
Bioinformatic analysis of the DEGs obtained from three mRNA expression profiling datasets. (a) Analysis of the DEGs in the three mRNA expression profiling datasets using the GEO2R tool. (b and c) Biological processes of DEGs related to IPF. (d) KEGG pathways obtained from the DEGs.

The GSE32537, GSE53845, and GSE10667 datasets were screened using the GEO2R tool to identify genes that were differentially expressed between the experimental and control groups, and 428, 661, and 1287 differentially expressed genes were identified, respectively. A total of 67 differentially expressed genes were found that exhibited exactly the same expression trends in all three microarray sets (the list of the 67 differentially expressed genes is shown in Supplementary Table 1). Of the 67 genes, 10 were downregulated in IPF and 57 were upregulated (Figure 2(a)).

**Figure 3:**
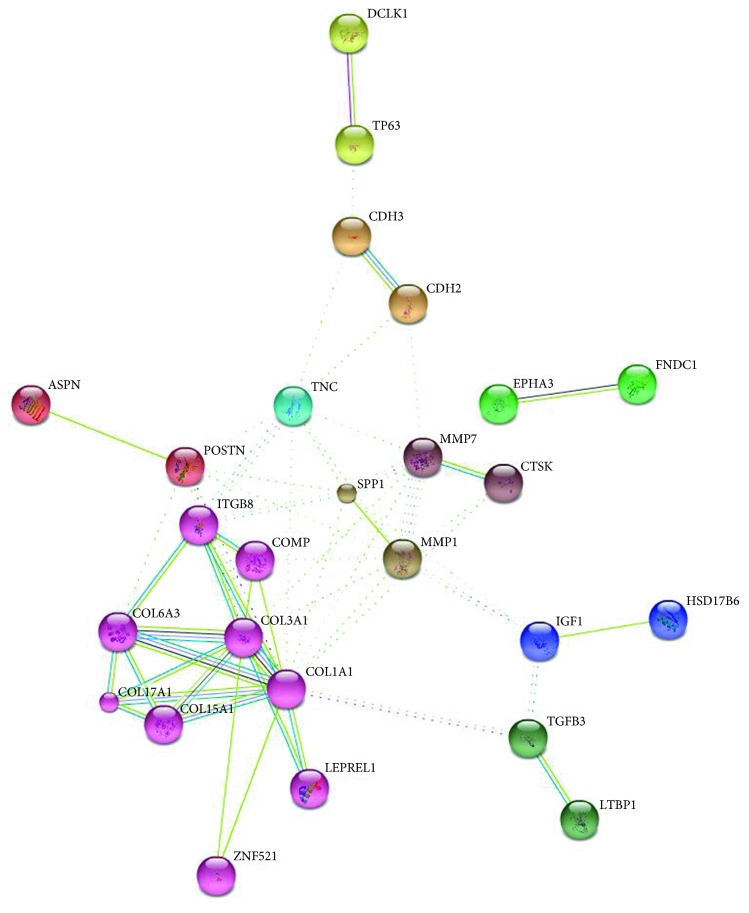
Protein-protein interaction network of DEGs acquired from STRING 9.1.

To reveal the biological significance of the common differentially expressed genes in the regulation of IPF at the unitary level, biological pathway enrichment and biological process annotation were performed on the above-described 67 genes using DAVID. Among the 81 biological processes, cell adhesion, biological adhesion, and skeletal system development were found to be significantly related to IPF regulation (*p* < 0.05) (Figures 2(b) and 2(c)). Based on KEGG pathway analysis, the enriched target genes were involved in the focal adhesion signaling pathway and ECM-receptor interact signaling pathway (Figure 2(d)). The relationship among the 67 common differentially expressed genes in IPF was further demonstrated using the STRING web tool. Overall, 57 interactions existed among the 67 proteins/genes in the PPI network (Figure 3). The connectivity degree of each node was calculated, and the top nine nodes with degrees ≧5 were COL1A1, MMP1, COL3A1, TNC, SPP1, MMP7, POSTN, ITGB8, and COL6A3. COL1A1, which had the highest degree [14] in the network, was considered the hub node because it interacted with many proteins, including LEPREL1, ZNF521, TGFB3, COL15A1, COL17A1, and TNC.

### 3.4. Analysis of the Correlation between Differentially Expressed MicroRNAs and Differentially Expressed Genes Associated with IPF

**Figure 4:**
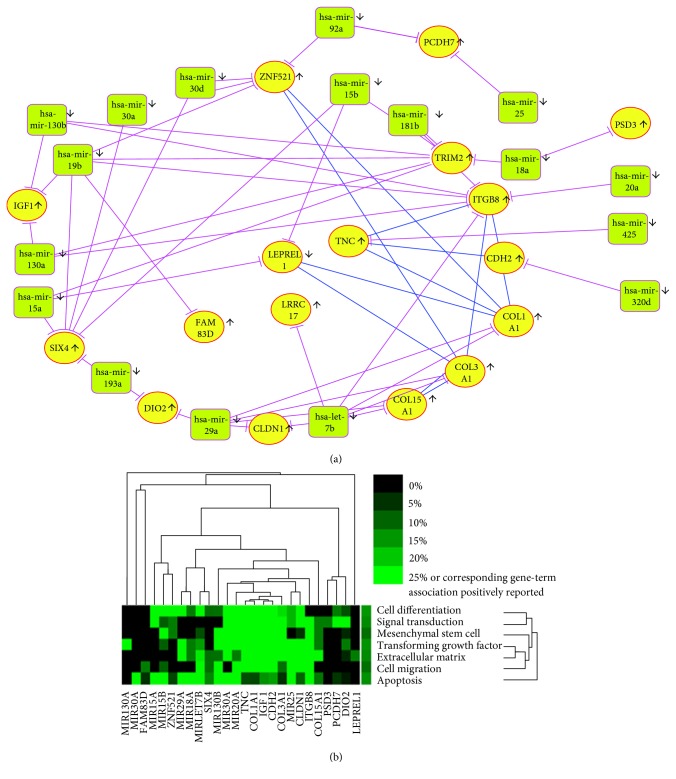
The regulation network of DE-miRNAs and DEGs in IPF. (a) The network of regulation of DE-miRNAs and DEGs in IPF. (b) Text mining of the DE-miRNAs and DEGs used GenCLip 2.0 software.

The candidate target genes of the 45 dysregulated microRNAs were predicted using microRNA-target interaction tools, including miRanda/mirSVR, targetScan, picTar, RNA22, and PITA. Genes identified by at least three prediction tools were selected as candidate targets, and intersections between the candidate target genes and the 67 common differentially expressed genes in the three microarray datasets were determined. Seventeen microRNAs and their target genes existed in the DEGs of the three common mRNA datasets (Supplementary Table 2). TRIM2 was predicted as the target of 7 microRNAs, and SIX4 and ITGB8 were predicted as the target of five microRNAs. There are totally 17 DEGs of three common mRNA datasets predicted can be regulated by dysregulated miRNAs, and 8 of the 17 have interactions in the PPI network (Supplementary Table 3). To further elucidate the correlations between miRNAs and potential target genes, miRNA-gene network analyses were generated by Cytoscape (Figure 4(a)). Text mining of the 17 DE-miRNAs and 17 DEGs was performed using the GenCLip 2.0 software. The occurrence frequency of terms in corresponding gene-related literature including cell differentiation, signal transduction, mesenchymal stem cells, transforming growth factor, extracellular matrix, cell migration, and apoptosis are shown in Figure 4(b). All of the above biological processes are significantly related to IPF.

## 4. Discussion

IPF is a chronic fibrotic lung disease that is characterized by increasing fibroblast proliferation and activation, including fibroblast accumulation, collagen synthesis, and deposition of extracellular matrix proteins and glycoprotein [28, 29]. MicroRNAs are a class of noncoding small RNAs that are approximately twenty-two nucleotides in length and are important regulators in gene regulation. The endogenous 19–25 nt noncoding RNAs can bind to the 3′-untranslated region (3′-UTR) of specific genes to inhibit the translation of the corresponding mRNA. Previous studies have shown that the pathogenesis of pulmonary fibrosis is related to various factors, including DE-miRNAs, DEGs, and microRNA-controlled differential gene expression [30, 31]. Therefore, screening for and identifying microRNAs and genes that are differentially expressed in IPF and investigating the correlations between DE-miRNAs and DEGs may shed light on the molecular mechanisms underlying IPF pathogenesis and provide guidance both for clinicians and for predicting prognosis.

GEO2R is an R programming language-based analytical tool that is used for studying DEGs. In the present study, microRNA expression microarray data from IPF and nondiseased control lung tissue samples (GSE32538) were analyzed, and 45 differentially expressed microRNAs were identified. Forty-two of these microRNAs were downregulated, whereas 3 microRNAs (hsa-mir-205, hsa-mir-34c, hsa-mir-31) were upregulated. Among the top 20 signaling pathways regulated by the 45 identified DE-miRNAs and 67 common DEGs, at least three are related to the progression of IPF, including cell cycle [32–34], TGF-beta signaling pathway [35–37], and adherens junction [38]. These results indicate that the 45 DE-miRNAs may be associated with the progression of IPF.

In lung epithelial cells and fibroblasts, the miRNAs could affect fibrogenic activity via targeting TGF-beta signaling events, *α*-Smooth muscle actin (*α*-SMA) and collagen, type I (COL1) gene expression. TGF-*β* is secreted for storage in the extracellular milieu, and it is kept inactive in a latent form by binding to latency-associated peptide (LAP). Released from LAP, TGF-*β* dimers sequentially associate with two primary transmembrane receptors, the type II and then the type I TGF-*β* receptor, activating receptor heterodimerization. Various signaling pathways and biological progresses are thereby initiated, including the Smad-mediated and non-Smad-mediated pathways, differentiation, proliferation, migration, epithelial mesenchymal transition (EMT), and ECM. Previous studies have shown that miRNAs are involved in regulating target genes in lung inflammation, EMT, ECM, and lung fibrosis processes [31].

Recent reports have shown that let-7 participates in pulmonary fibrosis by regulating EMT and TGF–*β* signaling activity. The let-7 miRNA family was first discovered [39] and extensively studied in metastasis. Its expression was significantly decreased in IPF lungs compared to normal lungs [30, 40]. Pandit et al. [40] elucidated that let-7d expression may be inhibited by TGF- *β*1, which is mediated by combination of SMAD3 with the let-7d promoter. Prior studies also showed that let-7d significantly downregulated the expression of HMGA2, SLUG, ID1, and ID2 in human primary fetal lung fibroblasts [11, 40].

Furthermore, miR-29 regulates a large number of genes associated with fibrosis. In the lung biopsies of patients with IPF, Montgomery et al. showed a significant decrease in the levels of miR-29a, miR-29b, and miR-29c as well as reduced trichrome staining in miR-29b mimic-treated mice in comparison with that in bleomycin-treated mice [41]. MiR-92a occupies an important role in pulmonary fibrosis. MiR-92a could decrease TGF-*β*1-induced Wnt1 inducible signaling pathway protein 1 (WISP1) protein expression (qRT-PCR and ELISA) in lung fibroblasts ex vivo [6].

Thus, in spite of the above specific profibrotic and antifibrotic miRNAs, the function of the rest of the DE-miRNAs remains to be clearly demonstrated.

Three sets of mRNA expression profiles were analyzed and a total of 67 genes were identified as DEGs in IPF. Enrichment analysis of biological processes and signaling pathways showed that the above-described 67 differentially expressed genes are significantly related to a series of biological processes such as cell adhesion, biological adhesion, and skeletal system development. ECM-receptor interaction and focal adhesion are significant pathways enriched by the DEGs that have been proven to be closely related to the regulation of IPF [42–44]. A previous study has demonstrated that cell adhesion and biological adhesion are the critical steps leading to the occurrence of pulmonary fibrosis [45–48]. These findings revealed that the 67 genes are significantly differentially expressed in all three IPF gene expression profile datasets and may be involved in the progression of IPF by participating in processes such as cell adhesion, biological adhesion, ECM-receptor interaction, and focal adhesion.

The epigenetic regulation of microRNAs plays an important role in the progression of IPF. MicroRNAs are reported to be one of the important mechanisms in pulmonary fibrosis. Therefore, in the present study, microRNA-mRNA interaction analysis was conducted using microRNA and mRNA expression profiles to obtain additional information related to IPF. By analyzing interactions between DE-miRNAs and DEGs in IPF and control tissues, we discovered that seventeen microRNAs may have regulatory effects on nearly half of the 45 identified genes. These findings indicate that the DE-miRNAs and DEGs described above may act in concert to participate in IPF. A total of 15 DE-miRNAs and 11 DEGs involved in this microRNA-target relationship have been identified by GenCLip 2.0 to be associated with cell differentiation, signal transduction, mesenchymal stem cells, extracellular matrix, cell migration, and apoptosis. At the same time, we analyzed the 67 DEGs in our manuscript with the clinical characteristics according reference [14]. As shown in Supplementary Table 1, there are several dysregulated transcripts associated with age (ITLN2, BTNL9, TNC, TDO2, SPP1, PSD3, and LTBP1); gender (SLC6A4, HSD17B6, CRTAC1, TNC, TDO2,SPP1, and LRRC17); and smoking (CFH, CDH2). Expression of cilium genes appears to identify two unique molecular phenotypes of IPF/UIP [14].The different molecular profiles may be relevant to therapeutic responsiveness in patients with IPF/UIP. There are 8 out of 10 downregulated DEGs that are downregulated in Group II compared with those in Group I, namely VIPR1, SLC6A4, NECAB1, LEPREL1, ITLN2, HSD17B6, HHIP, and CRTAC1 and 14 out of 57 upregulated DEGs that are upregulated in Group II compared with those in Group I, namely TRIM2, TP63, TMPRSS4, SPP1, SIX4, MMP1, ITGB8, GOLM1, CP, COL17A1, CLIC6, CDH3, CD24, and C12orf75.

These results indicate that the microRNA-target network constructed from 17 DE-miRNAs and 17 DEGs might shed new light on potential biomarkers for the prediction of IPF progression. The exact roles of these DE-miRNAs/DEGs will require further in-depth study.

## Supplementary Material

Supplementary Fig 1. Heatmap of pathway intersection (with 2 or more miRNAs) of De-mirRNAs. Supplementary Table1. 67 differentially expressed genes and their associated with clinical parameters and patient subgroups. Supplementary Table 2. 17 microRNAs and their target genes existed in DEGs of three common mRNA datasets. Supplementary Table 3. The 17 differentially expressed genes that can be regulated by dysfunctional miRNAs of GSE32538 datasets.

## Figures and Tables

**Table 1:** microRNA and gene expression microarray datasets related to idiopathic pulmonary fibrosis.

	Accession number of the dataset	Organization name	Platform	Status	Organism	Experiment type	Disease type	
							IPF	Control
MicroRNA	GSE32538 [14]	University of Colorado	GPL8786	Public on June 21, 2013	*Homo sapiens*	Noncoding RNA profiling by array	106	50
mRNA	GSE32537 [14]	University of Colorado	GPL6244	Public on June 21, 2013	*Homo sapiens*	Expression profiling by array	119	50
	GSE53845 [15]	Genentech, Inc.	GPL6480	Public on Oct. 14, 2014	*Homo sapiens*	Expression profiling by array	40	8
	GSE10667 [16–18]	University of Pittsburgh	GPL4133	Public on Feb. 20, 2009	*Homo sapiens*	Expression profiling by array	23	15

**Table 2:** Top 10 logFc of differentially expressed miRNAs obtained from the GSE32538 dataset.

Dysfunction	miRNA	logFc	adj. *p* value
Upregulated	hsa-miR-205	1.8093192	2.79E-08
	hsa-miR-34c-3p	2.2856359	1.91E-08
	hsa-miR-34c-5p	2.3310863	3.36E-07
	hsa-miR-31	2.3422275	1.28E-08

Downregulated	hsa-miR-532-5p	−1.9021268	5.03E-18
	hsa-miR-652	−1.8713939	8.44E-15
	hsa-miR-130a	−1.6770333	5.48E-11
	hsa-miR-210	−1.6699558	5.39E-11
	hsa-miR-500	−1.6698889	3.16E-15
	hsa-miR-193a-5p	−1.6402524	4.57E-15
