# Supplementary material for: Analysis of Microarray-Identified Genes and MicroRNAs Associated with Idiopathic Pulmonary Fibrosis
Source: Mediators Inflamm. 2017 May 14;2017:1804240. doi: 10.1155/2017/1804240 (PMC5446886; doi:10.1155/2017/1804240)
Supplement: Supplementary file 1 — Supplementary Fig 1. Heatmap of pathway intersection (with 2 or more miRNAs) of De-mirRNAs. Supplementary Table1. 67 differentially expressed genes and their associated with clinical parameters and patient subgroups. Supplementary Table 2. 17 microRNAs and their target genes existed in DEGs of three common mRNA datasets. Supplementary Table 3. The 17 differentially expressed genes that can be regulated by dysfunctional miRNAs of GSE32538 datasets. [file 1804240.f1.pdf]

Supplementary Fig 1. Heatmap of pathway intersection (with 2 or more miRNAs) of DE-miRNAs

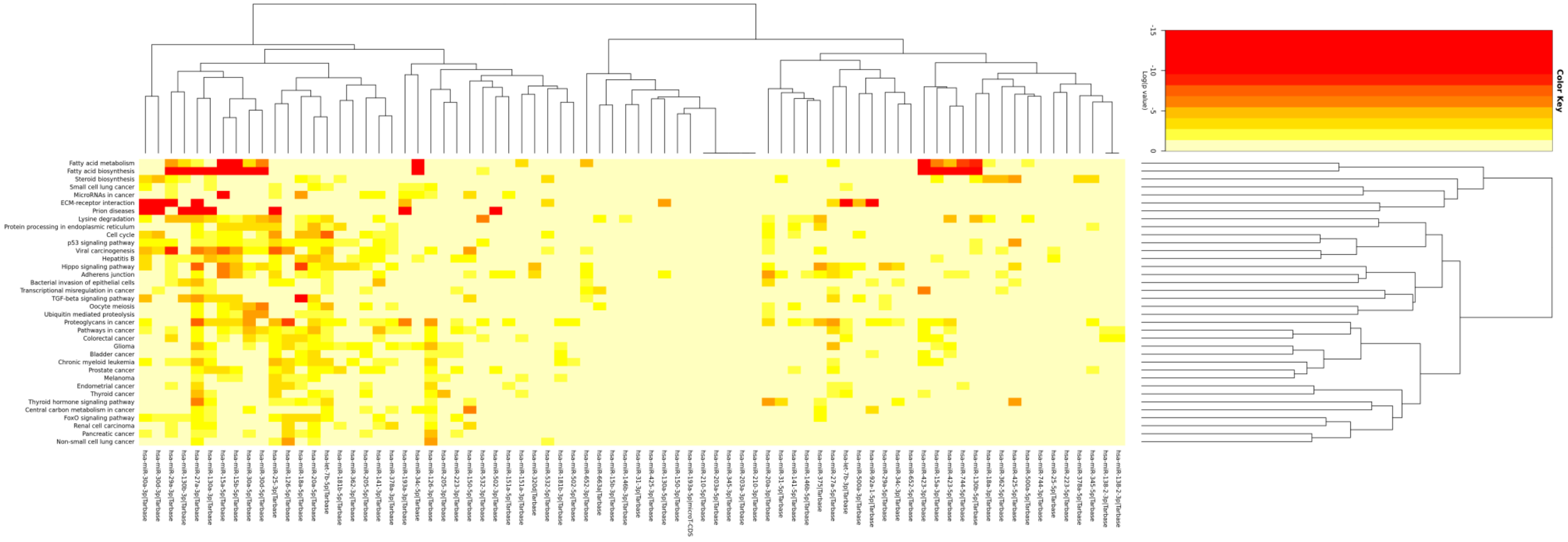

Supplementary Table1. 67 differentially expressed genes and their associated with clinical parameters and patient subgroups

Gene name	Gene ID	p-value(Age)*	p-value(Gender)*	p-value(Smoking)*	two-tailed tttest p-value(ClusterⅡ/Ⅰ)#	fold change(ClusterⅡ/Ⅰ)#
VIPR1	7433	0.126177	0.136686	0.344265	1.17E-15	-2.49
SLC6A4	6532	0.385541	0.041142	0.118514	2.08E-17	-5.47
NECAB1	64168	0.685413	0.332522	0.739776	2.20E-08	-1.56
LEPREL1	55214	0.654459	0.263689	0.322482	1.20E-14	-2.1
ITLN2	142683	0.006581	0.244348	0.616338	4.51E-08	-1.44
HSD17B6	8630	0.206396	0.029763	0.31042	3.74E-05	-1.56
HHIP	64399	0.241573	0.068495	0.13725	3.20E-12	-2.3
GALNT18	374378	/	/	/	/	/
CRTAC1	55118	0.444776	0.043385	0.0983701	0.000207	-1.37
BTNL9	153579	0.019168	0.112721	0.636179	/	/
ZNF521	25925	0.142374	0.828389	0.39197	/	/
TRIM2	23321	0.794137	0.625703	0.729964	6.12E-15	1.58
TP63	8626	0.774225	0.585081	0.855383	1.44E-22	2.43
TNC	3371	0.010034	0.016104	0.216074	/	/
TMPRSS4	56649	0.154469	0.219443	0.485892	6.85E-26	3.31
THY1	7070	0.177	0.186278	0.398847	/	/
TGFB3	7043	0.087113	0.322016	0.52223	/	/
TDO2	6999	0.014915	0.022895	0.390169	/	/
SULF1	23213	0.629003	0.160292	0.90913	/	/
SPP1	6696	0.031183	0.013325	0.863561	1.66E-17	6.4
SIX4	51804	0.600289	0.211946	0.955197	5.43E-28	2.27

SFRP4	6424	0.456407	0.626699	0.625528	/	/
SFRP2	6423	0.625595	0.078456	0.339615	/	/
SERPIND1	3053	0.097422	0.608744	0.642825	/	/
PSD3	23362	0.013523	0.927144	0.156792	/	/
POSTN	10631	0.63572	0.223909	0.723659	/	/
PCDH7	5099	0.185517	0.927286	0.511955	/	/
PAMR1	25891	0.592354	0.353737	0.363404	/	/
MXRA5	25878	0.870662	0.666444	0.375889	/	/
MMP7	4316	0.41145	0.303077	0.331552	/	/
MMP1	4312	0.078438	0.266318	0.349474	2.75E-15	5.46
LTBP1	4052	0.014039	0.543222	0.11248	/	/
LRRN1	57633	0.651246	0.936766	0.484772	/	/
LRRC17	10234	0.79711	0.013604	0.737823	/	/
LPPR4	9890	0.526718	0.622418	0.134065	/	/
LAMP5	24141	/	/	/	/	/
ITGB8	3696	0.246252	0.263522	0.608421	9.06E-16	1.68
IL13RA2	3598	0.060767	0.265732	0.658137	/	/
IGKC	3514	0.196328	0.749822	0.430495	/	/
IGK	50802	/	/	/	/	/
IGHV3-48	28424	0.306925	0.27175	0.325836	/	/
IGF1	3479	0.177857	0.053143	0.0738912	/	/
GOLM1	51280	0.369403	0.021218	0.58186	2.99E-24	1.86
FNDC1	84624	0.824688	0.538236	0.390894	/	/
FAM83D	81610	0.508339	0.059263	0.323836	/	/
EPHA3	2042	0.054568	0.014852	0.831154	/	/
DIO2	1734	0.41374	0.001194	0.136475	/	/

DCLK1	9201	0.668455	0.2099	0.93869	/	/
CXCL14	9547	0.115706	0.827791	0.463199	/	/
CTSK	1513	0.227509	0.602957	0.729709	/	/
CP	1356	0.713253	0.037292	0.942669	1.59E-25	4.29
COMP	1311	0.464452	0.411721	0.842376	/	/
COL6A3	1293	0.224806	0.083998	0.592143	/	/
COL3A1	1281	0.122769	0.062236	0.683391	/	/
COL1A1	1277	0.131306	0.057385	0.867915	/	/
COL17A1	1308	0.273684	0.618873	0.792419	7.36E-16	1.84
COL15A1	1306	0.483959	0.368165	0.215703	/	/
CLIC6	54102	0.231958	0.301658	0.974105	4.12E-23	2.49
CLDN1	9076	/	/	/	/	/
CFH	3075	0.083555	0.19109	0.0400911	/	/
CDH3	1001	0.009559	0.448395	0.411104	8.01E-10	1.6
CDH2	1000	0.46148	0.289592	0.0335855	/	/
CD24	100133941	0.2313	0.152439	0.154109	1.44E-15	2.12
CCDC80	151887	0.51957	0.600835	0.561529	/	/
C12orf75	387882	0.157218	0.773873	0.755695	2.67E-01	1.59
ASPN	54829	0.008958	0.91816	0.0741792	/	/
IGKV3-20	28912	/	/	/	/	/

* Data came from reference(14) Supplemental Table S2. Transcripts that differentiate IPF/UIP from controls at 5%FDR
# Data came from reference(14)Supplemental Table S3. Transcripts in cluster A with statistics for expression levels in clusters I and II.

Supplementary Table 2. 17 microRNAs and their target genes existed in DEGs of three common mRNA datasets

microRNA	LogFc	Target					
hsa-let7b-5p	-1.0305441	CLDN1	COL15A1	COL1A1	COL3A1	ITGB8	LRRC17
hsa-mir-29a-3p	-1.2426434	CLDN1	COL15A1	COL1A1	COL3A1	DIO2	
hsa-mir-19b-3p	-1.285607	TRIM2	SIX4	ZNF521	IGF1	ITGB8	FAM83D
hsa-mir-15a-5p	-1.274762	TRIM2	SIX4	LEPREL1			
hsa-mir-15b-5p	-1.5444445	TRIM2	SIX4	LEPREL1			
hsa-mir-130-3p	-1.6770333	TRIM2	IGF1	ITGB8			
hsa-mir-130b-3p	-1.0770705	TRIM2	IGF1	ITGB8			
hsa-mir-181-5p	-1.1743572	TRIM2	ITGB8				
hsa-mir-18a-5p	-1.4527038	TRIM2	PSD3				
hsa-mir-30a-5p	-1.3923532	ZNF521	SIX4				
hsa-mir-30d-3p	-1.3825413	ZNF521	SIX4				
hsa-mir-92a-3p	-1.0715239	ZNF521	PCDH7				
hsa-mir-193a-3p	-1.6402524	SIX4	DIO2				
hsa-mir-20a-3p	-1.1021217	ITGB8					
hsa-mir-25-3p	-1.1558484	PCDH7					
hsa-mir-320d	-1.1324349	CDH2					
hsa-mir-425-5p	-1.147462	TNC					

Supplementary Table 3. The 17 differentially expressed genes that can be regulated by dysfunctional miRNAs of GSE32538 datasets.

Gene name	PPI-degree	miRNA	LogFc		
			GSE32537	GSE53845	GSE10667
COL1A1	15	2	1.7733242	2.0907325	3.1987429
COL3A1	11	2	1.6166588	1.8811475	2.9565792
TNC	8	1	1.4030198	1.8460075	1.2676364
ITGB8	6	6	1.1520938	1.295175	1.1381997
COL15A1	4	2	1.3335039	1.9484725	1.5684563
CDH2	3	1	1.2781973	1.351365	2.7343814
IGF1	3	3	1.0380336	1.3539825	2.8572972
ZNF521	2	4	1.0190914	1.274905	1.0913039
CLDN1	0	2	1.5182668	2.335575	1.6341026
LRRC17	0	1	1.5244737	1.5028925	2.1294655
DIO2	0	2	1.2931864	2.757095	2.9104809
FAM83D	0	1	1.226981	1.47853	2.5953808
SIX4	0	6	1.1132993	1.26075	2.8027957
TRIM2	0	7	1.0588409	1.116125	1.2121226
LEPREL1	0	2	-1.155286	-1.477928	-1.194345
PSD3	0	1	1.0770657	1.0512575	1.1602056
PCDH7	0	2	1.0227611	1.1901525	1.3005977
